# 363. Autoimmune Sensory Neural Hearing Loss Apparently Triggered by Neurocysticercosis

**DOI:** 10.1093/ofid/ofac492.441

**Published:** 2022-12-15

**Authors:** Evan Davies, Alidad Arabshahi, Hamza A Lateef

**Affiliations:** All Care Family Medicine adn Urgent Care, Lorton, Virginia; Centers for Advanced ENT Care, Woodbridge, Virginia; George Mason University, Manassas, Virginia

## Abstract

**Background:**

Meniere’s disease (MD) occurs when an abnormal amount of fluid causes increased pressure in the inner ear or labyrinth. People with Meniere's can experience symptoms including vertigo, tinnitus, hearing loss, and a feeling of fullness in the ear. Various triggers for MD have been recognized and range from smoking and alcohol consumption to recent viral illness, allergies and anxiety. We present the case of a 37 year old Hispanic male, who presented with dizziness and tinnitus, which progressed to include left sided sensorineural hearing loss and he was diagnosed with Meniere’s Disease, later found to be triggered by Neurocystercercosis.

**Methods:**

Case report based on chart review.

**Results:**

The patient's laboratory work up was positive for anti-HSP antibody and he had an abnormal electrocochleography, left greater than right. His vestibulonystagmogram was positive for both central and peripheral findings and bithermal caloric irrigations suggested abnormal peripheral function with a 57% weakness in the left ear.

The patient was also referred to rheumatology and neurology. Neurodiagnostic work up included an MRI which revealed a post inflammatory calcification in the right parietal lobe and patient was presumed to have neurocystercercosis. He was treated with steroids, betahistine, and a low salt diet for his MD.

**Conclusion:**

To our knowledge, this is the first reported case in the literature of Meniere’s disease that may have been triggered by or concurrent with neurocysticercosis. This highlights the importance of a thorough work up to look for neurologic comorbidity for patients presenting with Meniere’s disease.

Neurocystercercosis is typically seen developing countries and is caused by the larval form of the parasite *Taenia solium* infecting the central nervous system. It commonly presents with seizures and such a manifestation associated with Meniere’s disease has not been previously reported.

**Disclosures:**

**All Authors**: No reported disclosures.

